# In-Plant Assessment of Peruvian Pisco Distillates Using Infrared Sensing Technologies

**DOI:** 10.1021/acs.jafc.5c14436

**Published:** 2026-03-31

**Authors:** Yalan Wu, Beatriz Hatta-Sakoda, Victor Hugo Toledo-Herrera, Jorge Mellado Carretero, Luis E. Rodríguez-Saona

**Affiliations:** † Department of Food Science and Technology, 2647 The Ohio State University, 325 Parker Food Science and Technology Building, 2015 Fyffe Road, Columbus, Ohio 43210, United States; ‡ Food Technology Department, Faculty of Food Industries, 113018 Universidad Nacional Agraria La Molina, Lima 15024, Peru; § Departament d’Enginyeria Química, Escola Tècnica Superior d’Enginyeria Química, 152687 Universitat Rovira i Virgili, Tarragona 43007, Spain

**Keywords:** Peruvian Pisco, quality control, FT-IR, distillation fraction

## Abstract

A novel approach using portable Fourier transform infrared spectroscopy (FT-IR) with an attenuated total reflectance accessory was developed to screen the quality of Pisco distillates. Pisco samples (*n* = 188) were obtained from distilleries in Peru, and their quality was determined by gas chromatography. FTIR discriminated the three distillate fractions (head, body, and tail) of Pisco, identifying their alcohol fingerprint (3091–2660 cm^–1^ and 1190–821 cm^–1^) as contributing to the class differentiation. Partial least squares regression showed strong correlations (*R*_pre_ = 0.93–1.00) and low errors of prediction (RMSEP) in quantifying 6 key quality components (ethanol, methanol, acetaldehyde, acetic acid, furfural, and titratable acidity). This approach offers a scalable alternative to conventional empirical methods, enabling distilleries to make precise distillation cuts, reduce production costs, and ensure consistent product quality. This is the first report of a portable FT-IR system capable of in-line monitoring of key quality parameters in the spirit distilleries.

## Graphical abstract

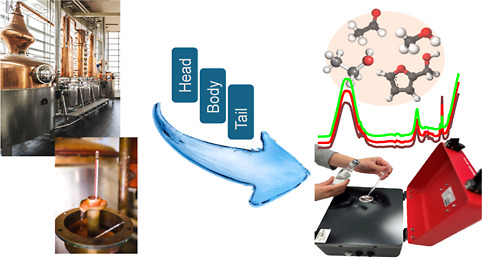

## 1 Introduction

The global market for alcoholic beverages was estimated at roughly $2.31 trillion in 2023, with projections indicating an expansion to about $5.72 trillion by 2032.^1–3^ In 2024, 11.7 million 9 L cases of Brandy/Cognac were sold in the U.S., generating $2.1 billion for distillers.^4^ One of the main challenges of the alcoholic beverage industry is to obtain quality products in an increasingly competitive market.^5–8^ In the case of spirits, adoption of new optical sensing strategies can improve quality, safety, and efficiency of the distillation process.^9^ We have selected Pisco, a grape brandy that stands as Peru’s signature national spirit,^10^ as our case study. It is uniquely produced through single-batch distillation in a copper still. Unlike similar grape-deviated spirits, it is neither rectified by adding water nor subjected to double distillation or aging.^11^ As a result, the distillation process is crucial for preserving its distinctive aroma.^9^ To optimize alcohol and volatile compound content, the distillate is collected in three consecutive fractions: head, body, and tail.^11^ The “head” is collected at the very beginning of the distillation, which has the highest alcohol content among the fractions and is characterized by undesirable levels of volatile acetaldehyde and esters.^12^ The “body” fraction is used for the final commercial product. With prolonged distillation time, the ethanol content gradually decreases, and the distiller makes the final “tail” cut before heavy fuel oils and other unwanted substances come off the still.^13^ The “head’ and “tail” are discarded due to compounds (acetaldehyde, methanol, furfural, and acids) that negatively impact sensory quality with unpleasant aromas and pose toxicity concerns.^14^ The chemical composition of Pisco is determined during distillation, therefore, recognizing the cut points during distillation is essential.^15^ A proper distillation cut optimizes the collection volume of “body” fractions for bottling and can help to correct errors from previous processes; however, improper cuts can introduce defects that are difficult to eliminate, negatively impacting the final product’s quality.^16^

Thus, quality management is crucial to ensure product consistency. According to the Peruvian regulation for Pisco’s designation of origin,^17^ the ethanol content must be between 38 and 48% ABV and tolerances are placed for total acidity (200 mg/100 mL acetic acid), superior alcohols (<350 mg/100 mL), and hazardous volatiles, such as methanol (<150 mg/100 mL), furfural (<5 mg/100 mL), and acetaldehyde (<60 mg/100 mL). Currently, distillation cuts are mostly determined through empirical methods based on the experience of distillers, who are also expert tasters, as they smell and taste to determine the cut of the distillates. The elevated concentrations of acetaldehyde and higher alcohols in the head fraction present a meaningful health concern for tasters, particularly with cumulative long-term exposure, even when spitting is practiced. Acetaldehyde is classified by regulatory authorities, including the U.S. Environmental Protection Agency, as a probable human carcinogen, based on animal evidence demonstrating tumor formation and documented acute and chronic toxic effects ranging from mucosal irritation to pulmonary edema and tissue necrosis at higher exposures. Growing mechanistic evidence further shows that acetaldehyde compromises DNA integrity, disrupts repair pathways, and alters normal cell-cycle regulation, thereby fostering conditions conducive to mutagenesis and carcinogenesis. These molecular insights align with epidemiological findings indicating that alcohol-related cancer risk is more strongly associated with cumulative acetaldehyde exposure than with ethanol itself, underscoring acetaldehyde’s central role in the pathogenesis of alcohol-related diseases.^11,18^ Regarding more parametric determinations of the cuts, vapor temperature or ethanol concentration has been utilized. Liebminger et al.^19^ showed that these methods do not work properly under most of the conditions. As a result, many studies have focused on adjusting specific operational strategies, such as reflux rate, evaporation rate, and dephlegmator temperature, to ensure the collection of a high-quality body fraction.^20,21^ Additionally, other research has explored different distillation systems to improve the reproducibility, sensory properties, and distribution of various volatile congeners.^22–24^ Tenorio et al.^25^ developed an algorithm based on the computational simulation to identify the distilling cuts, showing that the ratio of acetaldehyde to ethanol concentrations can determine whether the fermented musts meet cachaça’s legal standards. Furthermore, in-line conductivity measurements have been used to identify fraction cut points based on conductive compounds.^15,20^ However, these approaches are often tailored to specific distillation methods and may not be broadly applicable given the diversity of volatiles in different fractions and the variability in congeners among raw materials or even among cultivars of the same type. Additionally, these systems tend to respond slowly, limiting the process flexibility. Consequently, there remains a need for an objective method to determine cut points that are independent of both the type of spirit and the distillation technique used.^19^

Mid infrared spectroscopy is a nondestructive technique that, when combined with multivariate analysis, can provide unique chemical information on subtle compositional changes in spirits. The use of attenuated total reflectance (ATR) devices offers significant advantages, including minimal sample volume requirements, reduced preparation time, ease of instrument operation, high data reproducibility, and rapid analysis.^26–30^ Additionally, advances in hand-held and portable optical technologies for chemical detection have expanded the application of vibrational spectroscopy with enhanced accuracy outside the laboratory. These advancements have been driven by developments in solid-state lasers, optical components, wavelength selectors, and detectors that can be thermoelectrically air-cooled, achieving spectral resolution comparable to that of benchtop instruments.^2,31,32^ These portable systems are uniquely positioned for rapid on-site identification due to their speed, ruggedness, compactness, ease of use, and transportability.^33^

Fourier transform infrared spectroscopy (FT-IR), combined with pattern recognition analysis, has shown promising applications in the analytical determination of distilled alcoholic beverages, including ethanol content, methanol adulteration, aging duration, and brand authentication.^34–37^ Despite growing interest and recent advancements, no studies have reported a portable and real-time optical sensing approach for monitoring distillation processes and informing fraction cut decisions in spirits production. In this work, we address this gap by integrating a portable ATR–FTIR spectroscopic sensor with chemometric modeling to enable real-time and in-plant monitoring of the distillate composition and quality during distillation.

Our objective was to establish a rapid, nondestructive, and field-ready FT-IR method capable of monitoring key quality attributes of Pisco throughout the distillation process. We aimed to address the challenges faced by Pisco distilleries in making accurate and rapid on-site cut decisions, ensuring consistent product quality, reducing waste from head and tail fractions, and improving profit margins.

## 2 Materials and Methods

### 2.1 Samples

Pisco samples (*n* = 188) were kindly provided by Pisco 1615 Inc. (Pisco, Peru) and Mrs. Hatta-Sakoda from the University of La Molina (Peru). Pisco samples were obtained from seven traditional distilleries across the Ica region during the grape harvest seasons of 2023 to 2025. The data set comprises a diverse set of spirits produced from Quebranta (*n* = 68), Italia (*n* = 32), Albilla (*n* = 10), Moscatel (*n* = 3), Torontel (*n* = 7), and blended (*n* = 68) grape varieties and includes multiple distillation fractions (head *n* = 67, body *n* = 89, and tail *n* = 32). The division of the distillate into heads, bodies, and tails—commonly referred to as making “cuts”— was carried out by skilled master distillers. The master distillers determined these “cuts” by continuously evaluating the distillate’s aroma, flavor, texture, temperature, and alcohol strength by manually redirecting the flow into separate receivers as the character changes. Because cut points vary depending on equipment design, fermentation profile, and desired spirit style, they are not dictated by fixed timings but rely on the distiller’s sensory judgement and experience.

### 2.2 Determination of Major Quality Parameters Content

The reference values were measured based on the previous report^38^ with modifications. All samples were analyzed in triplicate with the mean values used for data analysis. The levels of methanol, ethanol, acetaldehyde, furfural, and acetic acid in Pisco distillates were measured based on an Agilent Technologies 6890 Gas Chromatograph (GC) equipped with a flame ionization detector (Santa Clara, CA, USA). Separation was performed on a 25 m × 320 μm (internal diameter) × 0.5 μm (film thickness) HP-FFAP column (Agilent Technologies, Santa Clara, CA, USA). A 1 μL of the sample was directly injected in the split mode at a ratio of 15:1, with a constant carrier gas flow of 24.3 mL/min, and an injection port temperature of 250 °C. The oven program started at 40 °C and was maintained for 4 min. The temperature then increased at a rate of 5 °C/min to 100 °C, followed by a rate of 10 °C/min to 160 °C. Finally, the temperature was raised to 220 °C at 55 °C/min and held for 2 min. External calibration curves were prepared using 99.9%-purity methanol, acetaldehyde, acetic acid, and furfural standards (Sigma-Aldrich, St. Louis, MO, USA) in 40% ethanol/water, covering concentration ranges of 0.75 to 153.4 mg/100 mL (methanol), 0.72 to 58.4 mg/100 mL (acetaldehyde), 4.4 to 355.8 mg/100 mL (acetic acid), and 1–16 mg/100 mL (furfural). Ethanol calibration standards ranging from 10 to 70% v/v were prepared by serial dilution. A 70% (v/v) stock solution was made by transferring 70.0 mL of ethanol into a 100 mL volumetric flask and diluting it to volume with deionized water. Subsequent standards were prepared by serially dilution of this stock solution with deionized water.

Methanol concentrations in the Pisco samples obtained from our collaborators exhibited a narrow distribution (4.8 to 50.9 mg/100 mL; standard deviation = 8.10). To enhance the applicability and robustness of our model across a broader range of sample compositions, we employed a spiking strategy to intentionally extend methanol concentrations to levels reported in economically motivated adulteration cases. To minimize potential matrix effects, spiking was performed using real distillate matrices rather than simplified solvent systems. Twenty Pisco samples were randomly selected and individually fortified with pure methanol. Each 100 mL sample was spiked once at a randomly assigned level between 50 and 70 mg, generating 20 additional samples. The methanol dose was varied for every sample to ensure that each spiked sample possessed a distinct concentration.

Titratable acidity (TA) of the Pisco samples was measured following the AOCS official method (942.15) using an automatic titrator (916 Ti-Touch, Metrohm AG, Ionenstrasse, Switzerland). TA values were reported as milligrams of acetic acid per 100 mL of Pisco. Because of the limited sample volume, measurements were performed on a total of 96 samples.

### 2.3 Collection of FT-IR Spectra

Spectral data were collected simultaneously with the measurement of reference values. FT-IR spectra were obtained using a portable 4500 series FT-IR spectrometer (Agilent Technologies, Santa Clara, CA, USA) controlled by MicroLab PC software (Agilent Technologies, Danbury, CT, USA). The instrument has dimensions of 22 × 29 × 19 cm and a weight of 6.8 kg and is combined with a triple-reflection ATR diamond crystal (2 mm in diameter, 200 μm effective area) along with a thermoelectrically cooled deuterated triglycine sulfate (dTGS) detector. For analysis, less than 10 μL of each sample was applied to the ATR crystal surface, and spectra were acquired in the 4000–650 cm^–1^ spectral range at a resolution of 4 cm^–1^. To minimize spectral variability caused by evaporation and the temperature dependence of alcohol–water mixtures, we continuously monitored the instrument temperature during data acquisition and applied consistent equilibration and handling procedures at the ATR crystal for all samples. A background spectrum was collected for each sample to correct for environmental fluctuations. For every sample, three replicate spectra were recorded and averaged for subsequent analysis. Between measurements, the ATR crystal was thoroughly rinsed with distilled water and dried with a cotton cloth.

### 2.4 Chemometrics and Multivariate Analysis

Spectral analysis was performed using multivariate statistical software (Pirouette version 5.0, Infometrix Inc., Woodville, WA, USA). Supervised classification was performed using SIMCA, where samples were assigned to the body (class 1), head (class 2), or tail (class 3). The data set was transformed through PCA for dimensionality reduction, with the resulting components used to construct models for individual classes and to calculate confidence intervals.^39^ The discriminating power plot produced by SIMCA highlighted the key infrared bands most relevant to distinguishing between the sample classes. Sample clustering was visualized by projecting the original data onto the PCA axes using a score plot.^40^ Classes were considered distinct from each other if the interclass distances exceeded 3.^41^ The interclass distance formula is as below

$$d_{ij}=\sqrt{\frac{d_{i\left(j\right)}^{2}+d_{j\left(i\right)}^{2}}{2}}$$

where *d*_*i*(*j*__)_^2^ is the squared distance of class *i* samples to model *j* and *d*_*j*(*i*__)_^2^ is the squared distance of class *j* samples to model *i*.

The spectral data were mean-centered and transformed by using a second-derivative Savitzky–Golay polynomial filter with a 35-point window prior to subsequent analysis with SIMCA. SG filtering allows for suppression of scattering effects, improved resolution of overlapping peaks, and amplification of minor spectral features.^42^

The classification performance was evaluated using accuracy, sensitivity, and specificity. These metrics quantify the proportion of correctly classified instances out of all instances, the proportion of true positives among actual positive cases, and the proportion of true negatives among actual negative cases, respectively. The formulas are

$$accuracy=\frac{TP+TN}{TP+TN+FP+FN}\in \left[0,1\right]$$

$$sensitivity=\frac{TP}{TP+FN}\in \left[0,1\right]$$

$$specificity=\frac{TN}{TN+FP}\in \left[0,1\right]$$

where TP, TN, FP, and FN refer to the numbers of true positive, true negative, false positive, and false negative, respectively.

Partial least-squares regression (PLSR) was used to predict the key quality parameters of Pisco distillates by building quantitative models that combine spectral data with reference measurements.^43,44^ The data set was randomly divided into calibration and external validation sets. Prior to PLSR analysis, the spectral data were normalized using the sample 2 norm and transformed with a second derivative (Savitzky–Golay window of 25–35 points). Sample-2-norm (vector-length, l 2 normalization) scales each spectrum by its Euclidean norm so that differences in overall intensity do not dominate the models. The formula is

$$f_{i}={\left(\sum_{j}^{m^{*}}x_{ij}^{2}\right)}^{1/2}$$

where the element *x*_*ij*_ is the *j*th variable measurement on the *i*th sample, the symbol m* indicates included variables. Each included variable in the sample is then divided by *f*_*i*_.

80% of the samples were allocated to the calibration set, and the remaining 20% were reserved for external validation. Model performances were assessed by calculating the correlation coefficient of cross-validation (*R*_cv_), the root-mean-square error of cross-validation (RMSECV), the correlation coefficient of prediction (*R*_pred_), the root-mean-square error of prediction (RMSEP), along with outlier detection using sample studentized residual versus leverage.^45–47^ The optimal number of PLSR factors for each model determined based on RMSECV reaches its minimum value. RMSEP indicates the prediction error associated with independent or unknown sample concentrations. Outliers were identified and discarded from the models when samples exhibited substantial residuals, irregular behavior, or high leverage values. Model performance was evaluated using the residual predictive deviation (RPD), calculated as the standard deviation of the response variable divided by the RMSEP. The RPD was used to evaluate the quality of the model performances, with values lower than 1.9 being considered insufficient for most applications, between 2 and 2.4 indicated rough screening applications, between 2.5 and 2.9 models are adequate for screening, 3.0 to 3.4 models are suitable for quality control, 3.5 to 4.0 models are satisfactory for process control, and above 4.1 models are excellent for any application.^48^

## 3 Results and Discussion

### 3.1 Characterization of the Pisco Distillation Fractions

The GC-FID technique was employed to assess the key quality attributes of Pisco distillates (Figure A1). A summary of the reference analysis results for the major quality parameter profiles across the three distillation fractions is presented in Table 1. The level of ethanol and acetaldehyde was highest in the head fraction, followed by the body, with the lowest levels observed in the tail, a trend consistent with the findings reported by Tsakiris.^11,49^ We found that there was no significant difference (*P*-value = 0.09) in methanol levels between the head and body fractions, which in agreement with the Hatta’s finding.^11^ The ethanol levels of the Pisco samples in the body, the commercial fraction of the distillation process, ranged from 31.43% to 41.21%. We found that the ethanol content in 56 out of 107 body samples was below the regulatory range (38–48% ABV). Although hydrometry remains the commonly used procedure by industry for determining the alcoholic strength by volume (ABV), GC-FID provides a direct and highly accurate measurement of ethanol in distilled spirits. GC-based quantification offers superior precision and is not affected by user-dependent variability, temperature fluctuations, or calibration drift—factors that routinely compromise hydrometer accuracy. Hydrometer methods also require strict temperature correction to 20 °C and regular calibration under regulatory guidelines (e.g., TTB Gauging Manual; OIV procedures), and deviations from these requirements can introduce significant error. In contrast, validated GC-FID methods consistently demonstrate high selectivity, linearity, and low measurement uncertainty, underscoring their reliability as analytical approaches for ethanol determination. Similar to other findings,^50–52^ we showed that the head and body fraction of Pisco has a higher concentration of acetic acid compared with tails. This can be explained by the nature of Pisco batch distillation, where acetic acid separates based on its solubility in alcohol and water, thus, the higher alcohol in the head and body fractions will enhance the solubility of acetic acid which evaporates along with ethanol during distillation.^53^ Furfural, a toxic compound posing a risk to consumer health, was detected in 118 samples, with 14 out of 17 tail samples exceeding regulatory limits (5 mg/100 mL). It primarily appears at the end of the body and the beginning of the tail fractions during distillation, triggered by prolonged heating under acidic conditions.^54^

**Table 1:** Summary of Major Quality Parameters in Pisco of the Head, Body, and Tail Based on GC-FID Analysis^a^

parameters	fractions	*N*	min	aax	average	STD
acetaldehyde (mg/100 mL)	head	67	0.44	112.76	18.70	14.34
	body	87	0.50	14.43	3.58	3.29
	tail	26	0.05	8.81	1.30	1.88
methanol (mg/100 mL)*	head	66	6.59	50.92	22.91	8.62
	body	89	12.02	49.26	28.02	7.30
	tail	32	4.80	41.00	13.41	8.31
ethanol (%)	head	67	36.61	70.57	59.41	5.76
	body	89	31.43	41.21	36.60	3.05
	tail	32	7.61	17.14	12.13	2.20
acetic acid (mg/100 mL)	head	62	6.7	55.0	14.4	12.5
	body	90	5.6	81.4	28.2	15.4
	tail	32	1.0	16.2	5.7	3.2
TA (mg acetic acid/100 mL)	head	15	14.0	65.8	32.9	15.0
	body	65	7.7	77.4	32.0	16.1
	tail	16	5.8	9.7	5.8	2.4
furfural (mg/100 mL)	head	17	0.22	3.43	0.75	0.99
	body	84	0.32	4.10	1.62	0.86
	tail	17	3.39	6.31	5.33	0.82

a Note: the statistical results are based on data after removing outlier samples. *Methanol statistical results are only based on the pure Pisco samples, the 20 spiked samples are excluded.

Figure 1 presents a comparison of the average FT-IR spectra for the three distillation fractions. Water corresponded to characteristic bands observed at 3650–3000 cm^–1^ and 1646 cm^–1^.^26^ The bands range between 2980 and 2800 cm^–1^ was associated with the C–H and O–H stretch of alcohols, alkyl, and aldehydes. The small peak at 1450 cm^–1^ and 1275 cm^–1^ corresponded to C–OH bending deformation and C=O stretching in acids.^55^ Bands of 1087 and 1045 cm^–1^ indicate C–O stretching and C–H bending of alcohols, mainly by ethanol in Pisco. The band of 877 cm^–1^ was due to aromatic compounds in Pisco.^56^ The spectral profile of the head and body distillate fractions was similar but showed differences in the band intensities, especially associated with the water and fingerprint bands that indicated the higher alcohol and congeners contents in the head fractions. The tail fraction exhibits the highest water content and the least information in the fingerprint region, indicating more diluted samples.

**Figure 1:**
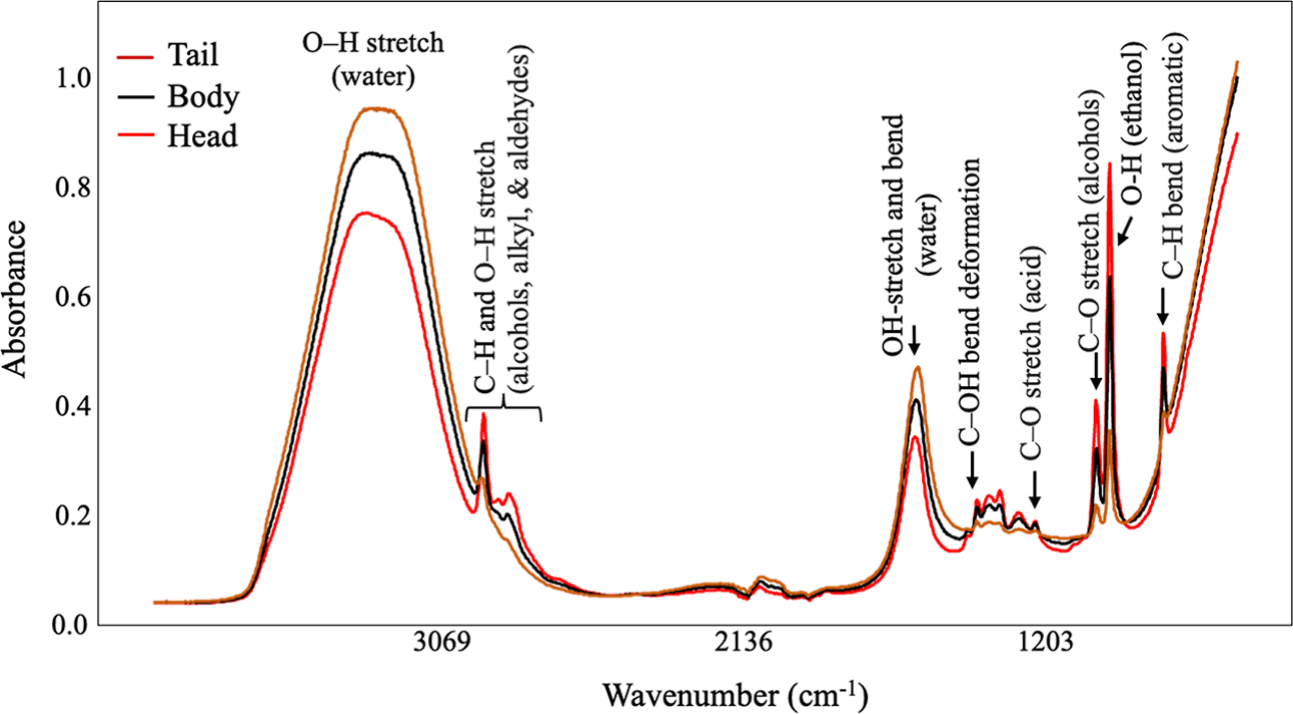
Comparison of the average FT-IR spectra representing the three fractions (head, body, and tail) of Pisco distillates.

### 3.2 SIMCA Classification for Pisco Distillates Identification

The SIMCA model for Pisco distillates was generated by using 150 samples (80% of all received samples) and selecting the spectral ranges at 3103–2665 cm^–1^ and 1527–832 cm^–1^ that contained the most unique fingerprinting information. For each class, the optimal number of latent variables (LVs) was chosen as the smallest number that minimized the SECV. Selecting an appropriate number of latent variables is critical for maintaining a balance between underfitting and overfitting, thereby supporting reliable model predictions. An insufficient number of components may fail to capture the underlying variance and result in underfitting, whereas incorporating too many descriptors can introduce irrelevant variation and ultimately cause the model to overfit.^44^ Robust classification required employing 3, 3, and 2 principal components for the head, body, and tail classes, explaining 99.8%, 99.9%, and 98.9% of the variance, respectively. The class projection plot of the training model (Figure 2A) displayed clear clustering patterns, forming three well-defined groups corresponding to distinct distillate fractions in a three-dimensional space. The interclass distance (ICD) ranged from 5.0 to 37.9 (ICD > 3) (Table 2), indicating substantial Euclidean distances between cluster centers, which is critical for accurate identification and differentiation.^57^ The discriminating power plot (Figure 2b) highlights the variables with the greatest impact on classification maximized intercluster differences and minimized intracluster differences.^58^ Bands in the 854–910 cm^–1^ range were found to be most important for distinguishing the three fractions, which can be attributed to the C–O bond of primary alcohols. The SIMCA model’s performance was evaluated using an independent external validation set including 14 head samples, 15 body samples, and 9 tail samples. All validation samples were correctly classified into their respective groups, except for 3 body samples that were not matched to any class. One of the unmatched samples was a unique Pisco product that was macerated with passion fruit, another contained the highest levels of acetic acid (∼200 mg/100 mL) among all Pisco, and last a Pisco sample that was collected early in the distillation (5 min) process. The classification model showed high accuracy (97.5%), sensitivity (>93.8%), and 100% specificity, which means the generated model is able to determine Pisco samples based on their corresponding distillation fraction.

**Figure 2:**
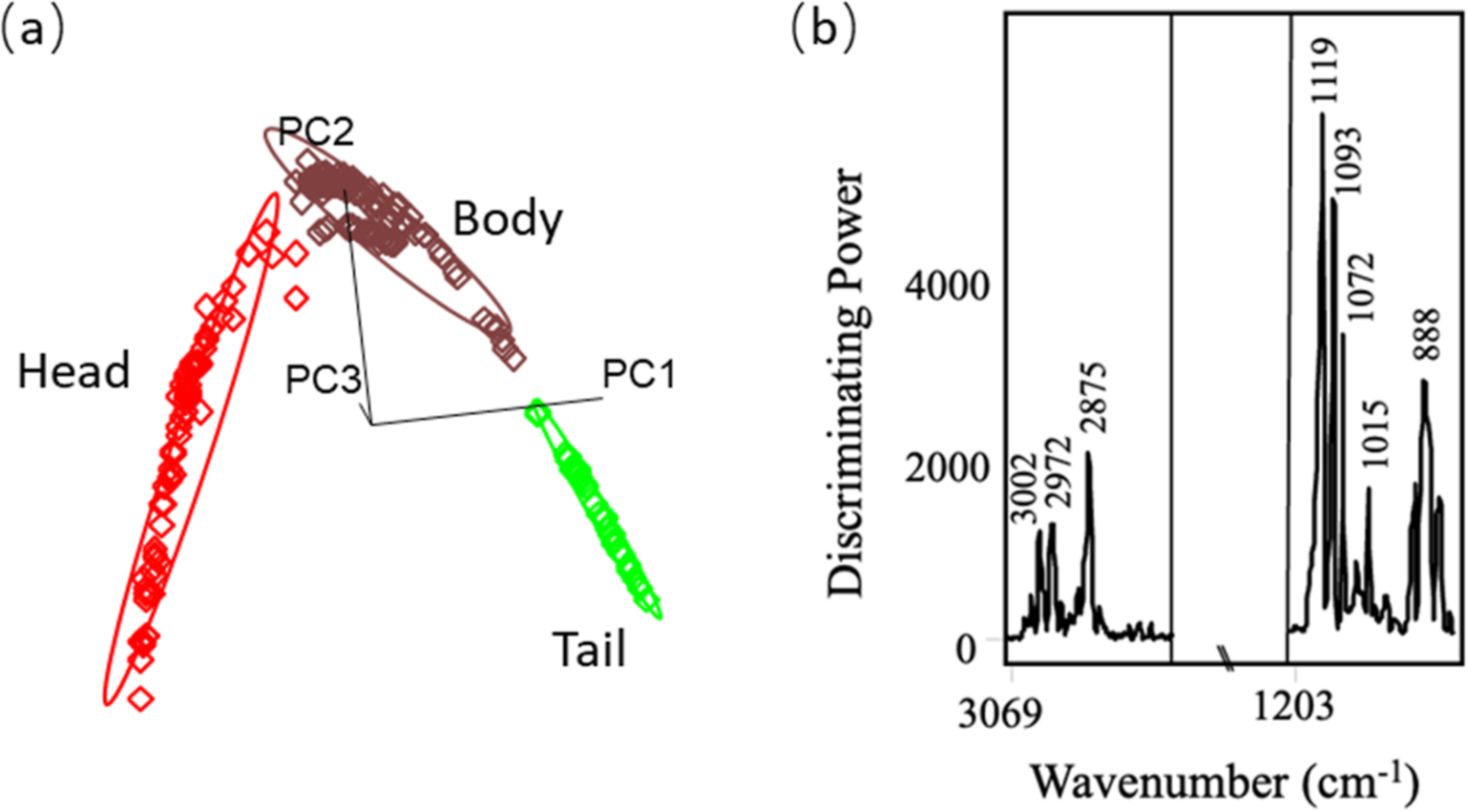
Class projection (A) and discriminating power (B) plots for Pisco head, body, and tail distillates classification based on SIMCA algorithm.

**Table 2:** Performance Statistics (Interclass Distance) of SIMCA Models Developed with Spectra Collected from Different Distillate Fractions of Pisco

	body@3	head @3	tail@2
body	0.00	-	-
head	5.01	0.00	-
tail	12.31	37.90	0.00

Our classification algorithm revealed potential incorrect categorization of samples in our training set. Samples labeled as “body” exhibited characteristics similar to tails and clustered within the tail group, four “head” samples were clustered with the body group, and one “tail” sample was grouped with the body. Upon conferring these misclassifications to our collaborators in Peru, they looked at their code information and confirmed that those samples were indeed misclassified when being bottled for shipment to our facilities and that the SIMCA algorithm correctly identified the nature of the samples.

### 3.3 Partial Least-Squares Regression Models

Key quality parameters in the distillation of Pisco (ethanol, acetaldehyde, methanol, furfural, acetic acid, and titratable acidity) were predicted by combining spectral data with the GC-FID reference values. All the samples were randomly divided into calibration and validation sets prior to PLSR analysis. The performance statistics of the developed PLSR models are presented in Table 3. The differences in sample numbers between the calibration and validation sets for different quality parameters are due to some samples falling below the detection limit and a few outliers being removed during model development based on leverage diagnostics.

**Table 3:** Statistical Performance of the PLSR Prediction Models for Key Quality Parameters in Pisco Distillates Developed Using Portable FT-IR Instrument

parameter	calibration model				validation model			
	*N*	factor	RMSECV	*R*_cv_	*N*	RMSEP	*R*_pre_	RPD
acetaldehyde (mg/100 mL)	141	6	2.37	0.98	37	2.23	0.96	3.19
methanol (mg/100 mL)	150	6	3.73	0.98	38	3.70	0.98	4.85
acetic acid (mg/100 mL)	136	7	7.74	0.96	34	8.03	0.97	5.98
ethanol (%)	148	3	1.60	1.00	37	1.33	1.00	11.57
TA (mg acetic acid/100 mL)	76	9	8.81	0.97	20	10.77	0.98	6.11
furfural (mg/100 mL)	91	4	0.57	0.95	23	0.55	0.93	3

PLSR models demonstrated the best performance when restricted to infrared regions relevant to the target parameter. Selecting distinct wavelengths enhanced prediction accuracy relative to the entire spectrum as it excluded variables that were irrelevant, noisy, or otherwise unreliable.^59^ During model development, the data were preprocessed using the second derivative and normalization by the sample 2-norm. The optimal number of factors, corresponding to the lowest SECV, varied between 3 and 6 which allowed the model to capture key variance without overfitting noise or underrepresenting the data’s variability.^60^ Table 3 shows the high accuracy of the models in predicting the various quality attributes of Pisco, with *R*_cal_ equal to or greater than 0.95, and low error (RMSECV) for furfural (5.7 ppm), acetaldehyde (23.7 ppm), methanol (37.3 ppm), acetic acid (77.4 ppm), TA (0.088 g acetic acid/L), and ethanol (1.6%).

Furthermore, the PLS regression vectors were used to identify the regions of the original spectra associated with the greatest variation in the calibration set (Figure 4), enabling the deduction of functional groups in Pisco distillates that are responsible for the observed variance in each quality parameter. Positive and negative bands in the regression vectors correspond to positive and negative correlations, respectively, while zero value implies no influence. Key bands affecting the ethanol content in Pisco were found by regression vectors ranged from 3071 to 2698 cm^–1^ and 1208–715 cm^–1^ which represents the stretching vibration of CH_2_, C–O, and O–H groups in ethanol (Figure 4A). Anjos et al.^26^ identified the ethanol content in grape-derived spirits as related to 1292–663 cm^–1^, in agreement with our finding. The regression vector for methanol (Figure 4B) showed the strong and characteristic band centered at 1015 cm^–1^ associated with C–O stretching vibration (2, 35). The regression vector for acetaldehyde displayed a major band at 950 cm^–1^ typically assigned to methyl rocking modes (Figure 4C).^61^ The regression vector for furfural (Figure 4D) confirmed the presence of the furan ring through a bond stretch at 1080 cm^–1^ and 1020 cm^–1^, while the band at 880 cm^–1^ corresponded to C–H in-plane bending.^62^ For titratable acidity and acetic acid (Figure 4E,F), the region between 1800 and 1134 cm^–1^ provided unique signals from organic acids, contributing the highest regression vector for building the prediction models. Specifically, the band at 1642 cm^–1^ (O–H bending vibration in water and O–H deformation), 1712 cm^–1^ (C=O stretching vibrations), and 1275 cm^–1^ (C–O stretching) indicated the presence of carboxylic acids.^63^

**Figure 3:**
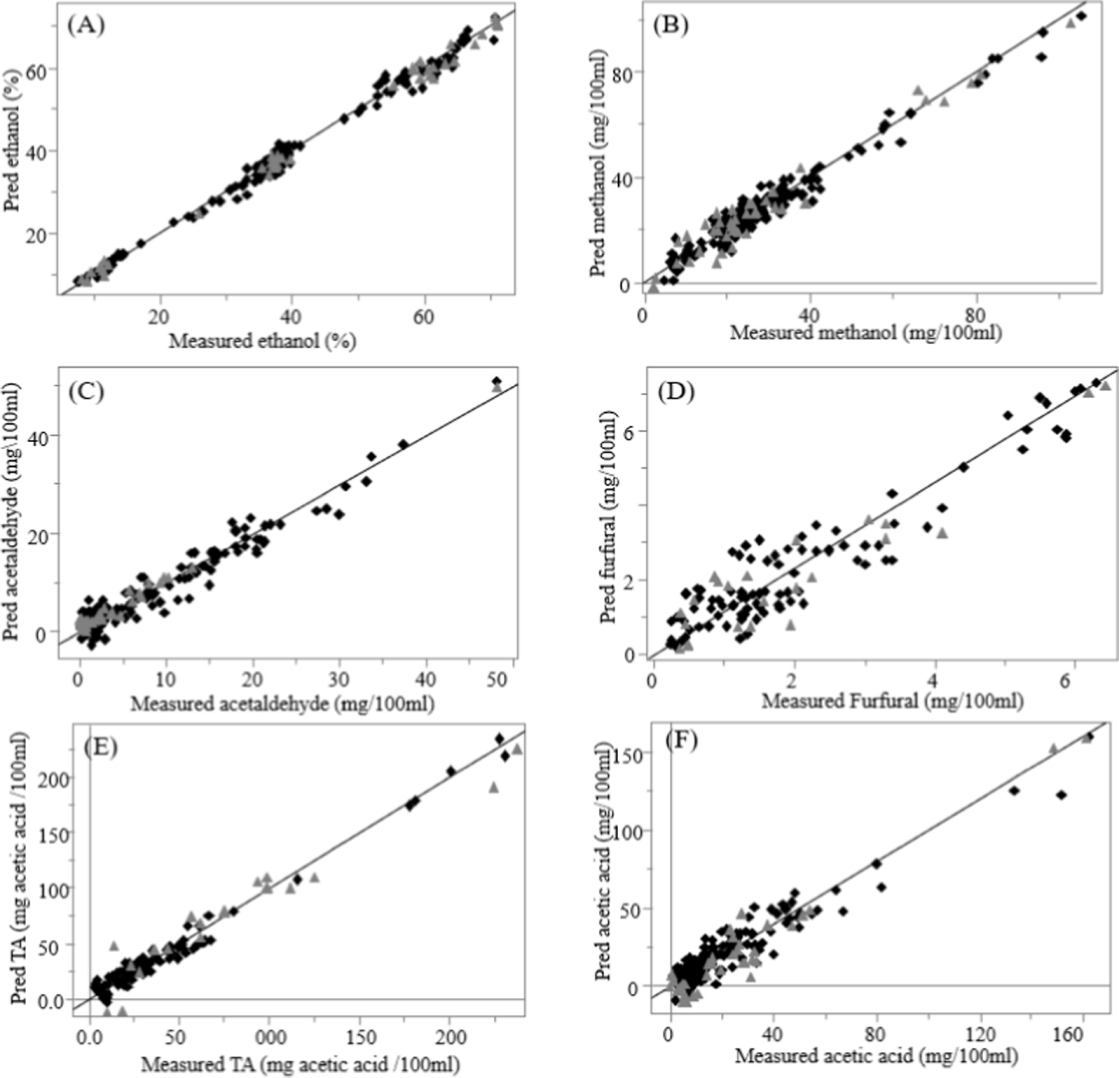
Partial least-squares regression (PLSR) correlation plot relating the FT-IR spectra and their corresponding quality parameter levels (black triangle: samples in the calibration set; gray square: samples in the external validation set).

**Figure 4:**
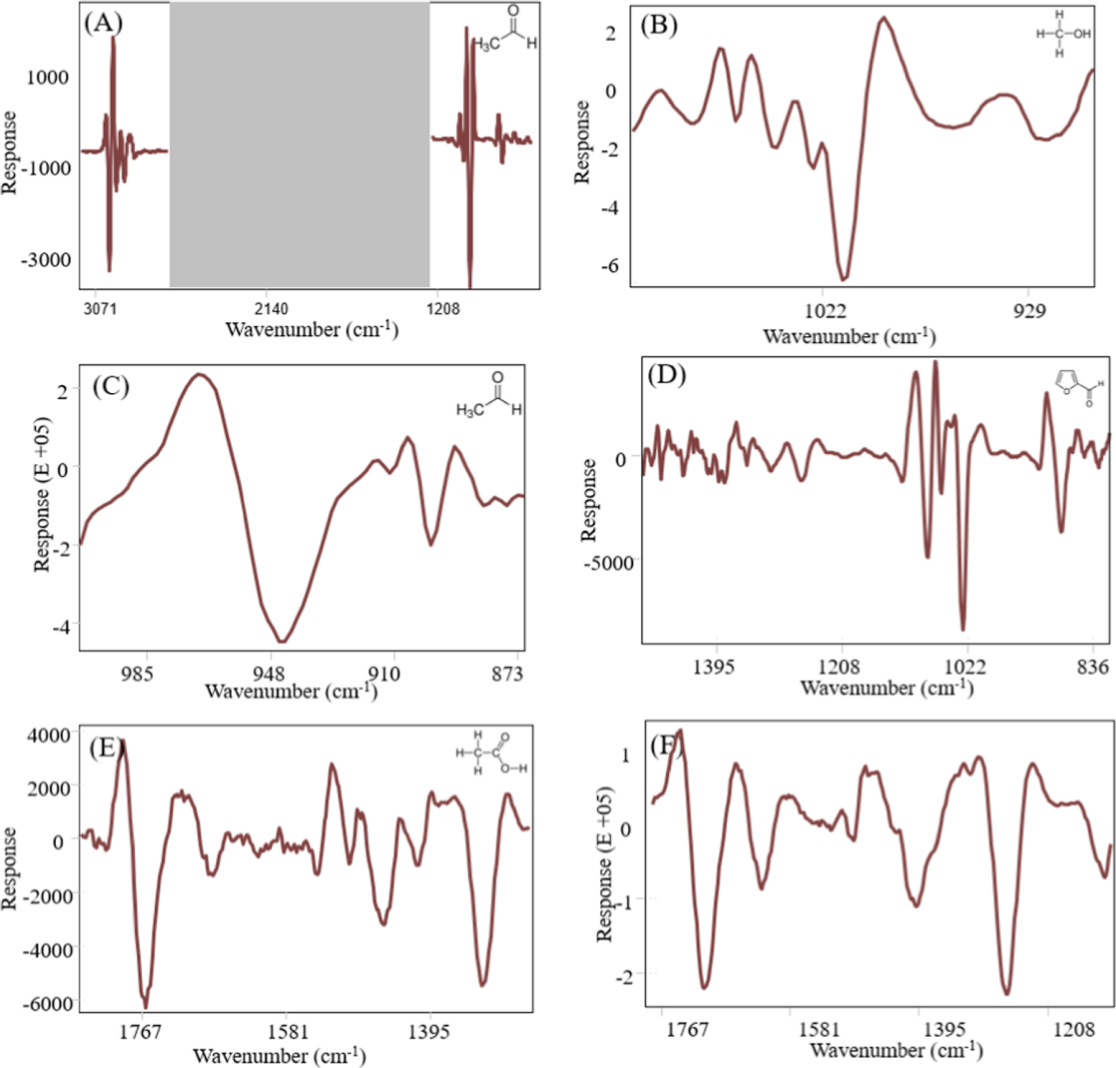
Regression vector plots of each PLSR model displaying the estimated weighted regression coefficients for (A) ethanol, (B) methanol, (C) acetaldehyde, (D) furfural, (E) total acidity, and (F) acetic acid.

The robustness of the calibration models was assessed through external validation with an independent sample set (Table 3). For each PLSR model developed, the cross-validation statistics (obtained through the leave-one-out approach) and external prediction results showed strong agreement, further confirming the robustness of the models. As illustrated by the correlation plots in Figure 3, the calibration and validation sets exhibited similar sample composition ranges and showed good fit between the predicted values by FT-IR and all six quality parameters from the Pisco distillates. Another important indicator for evaluating the performance of prediction models is the RPD. Ethanol, methanol, acetic acid, and titratable acidity all showed RPD values above 4.1, with ethanol reaching a maximum of 11.57, indicating excellent predictive performance and broad applicability. Acetaldehyde and furfural showed RPD values of 3.0 and 3.5, respectively, suitable for quality control. Furfural showed the lowest RPD value (3.00) likely because of its low concentrations (0.2 to 6.3 mg/100 mL) and the relatively low variance in the sample set (standard deviation of 1.64).

Table 4 summarizes previous studies that used FT-IR spectroscopy to monitor chemical indicators in distilled spirits. All of these investigations relied on benchtop FT-IR instruments equipped with either attenuated total reflectance (ATR) accessories or diffuse-reflectance sample holders. Notably, several reported models did not apply spectral preprocessing techniques that could have improved the model robustness. Although benchtop FT-IR systems have shown strong performance for ethanol and methanol screening,^27,33,64,65^ their limited portability restricts their suitability for on-site analysis. Additionally, comparison with the acetaldehyde prediction reported by Anjos et al.^26^—who analyzed grape-derived spirits using a benchtop FT-IR instrument with ATR and applied multiplicative scatter correction (MSC)—indicates that our model achieved lower prediction error despite working with lower analyte concentrations. Overall, the models developed in our study using a portable FT-IR device demonstrated comparable or superior predictive performance relative to those of these benchtop systems.

**Table 4:** Overview of Previous Studies Performed Using FT-IR Spectroscopy to Detect Quality Parameters in Distillated Spirits

spirits	quality parameters	content range	instrument	chemometrics	results	references
raki	methanol	0.5–10%	PerkinElmer benchtop with ATR	OSCW^a^	*R* = 0.98, RPD = 8.35 RMSEP = 0.35	^34^
	titratable acidity	-	PerkinElmer Spectrum 100 benchtop	1st & 2nd derivative^b^, WCS^c^	*R* = 0.73, RPD = 1.67, RMSEP = 0.32	^64^
spirit drinks	ethanol, methanol	ethanol: 25.0–78.1%, methanol: 0–1272 mg/100 mL	PerkinElmer Spectrum 100 benchtop	N/A	ethanol: *R* = 0.94, RMSEP = 0.35, methanol: *R* = 0.98, RMSEP = 36.9	^65^
brandy	acetic acid	0.07–0.28 g/L	MultiSpec benchtop	N/A	*R* = 0.94, RMSEP = 0.064	^66^
baijiu	acetic acid	-	PerkinElmer benchtop	N/A	*R* = 0.96, RMSEP = 3.11 mg/100 mL	^67^
mezcal	ethanol, methanol	ethanol: 10–50%, methanol: 10–50%	PerkinElmer GX benchtop	smoothing, 2nd derivative	ethanol: *R* = 0.99, RMSEP = 0.26, methanol: *R* = 0.97, RMSEP = 0.57	^68^
grape spirits	ethanol, methanol, acetaldehyde	ethanol: 34–45%, methanol: 30–2227 mg/100 mL, acetaldehyde: 17.5–261 mg/100 mL	Bruker benchtop (Alpha) with ATR	MSC^d^, SLS^e^, 1st & 2nd derivative	methanol: *R* = 0.99, RMSEP = 34.7, ethanol: *R* = 0.97, RMSEP = 0.36, acetaldehyde: *R* = 0.98, RMSEP = 10.4	^26^
Pisco	ethanol, methanol	ethanol: 21.1–43.8%, methanol: 18.1–45.2 mg/100 mL	Agilent Cary 630	mean-center, smoothing	ethanol: *R* = 0.96, RMSEP = 1, methanol: *R* = 0.88, RMSEP = 2.3	^35^

a OSCW: orthogonal signal correction in combination with wavelet.

b 1st & 2nd derivative: second derivative.

c WCS: wavelet compression of spectra.

d MSC: multiplicative scatter correction.

e SLS: straight line elimination.

Our study demonstrates that a portable ATR–FTIR device coupled with pattern-recognition models enables rapid, noninvasive analysis of Pisco distillates, providing results within 20 s and requiring no sample preparation. The optimized models achieved classification accuracies above 97.5% and strong PLSR validation performance (*R* ^2^ = 0.94–1.00) for six key quality parameters (ethanol, acetaldehyde, methanol, furfural, acetic acid, and titratable acidity). Supervised classification also flagged six factory-mislabeled samples, illustrating the utility of this approach for quickly identifying production issues. To the best of our knowledge, this is the first demonstration of monitoring distillate fractions during batch distillation using a portable ATR–FTIR platform. Collectively, these results support the feasibility of deploying ATR–FTIR for near-real-time, in-field quality screening and suggest that broader implementation could enhance process consistency while reducing analytical time and labor in distilled-spirits production.

## Supplementary Material



## Notes

Representative gas chromatograms of head, body, and tail of Pisco distillates (PDF)
