# Supplementary material for: In-Plant Assessment of Peruvian Pisco Distillates Using Infrared Sensing Technologies
Source: J Agric Food Chem. 2026 Mar 31;74(14):11753–62. doi: 10.1021/acs.jafc.5c14436 (PMC13088242; doi:10.1021/acs.jafc.5c14436)
Supplement: Supplementary file 1 [file jf5c14436_si_001.pdf]

In-Plant Assessment of Peruvian Pisco Distillates Using Infrared Sensing Technologies

Yalan Wu[1], *Beatriz Hatta-Sakoda*[2], *Victor Hugo Toledo-Herrera*[2], *Jorge Mellado Carretero*[3], *Luis E. Rodríguez-Saona*[1]*

[1] Department of Food Science and Technology, The Ohio State University, 325 Parker Food Science and Technology Building, 2015 Fyffe Road, Columbus, OH 43210, USA

[2] Food Technology Department, Faculty of Food Industries, Universidad Nacional Agraria La Molina, Lima 15024, Perú

[3] Departament d'Enginyeria Química, Escola Tècnica Superior d'Enginyeria Química, Universitat Rovira i Virgili, Tarragona 43007, Spain.

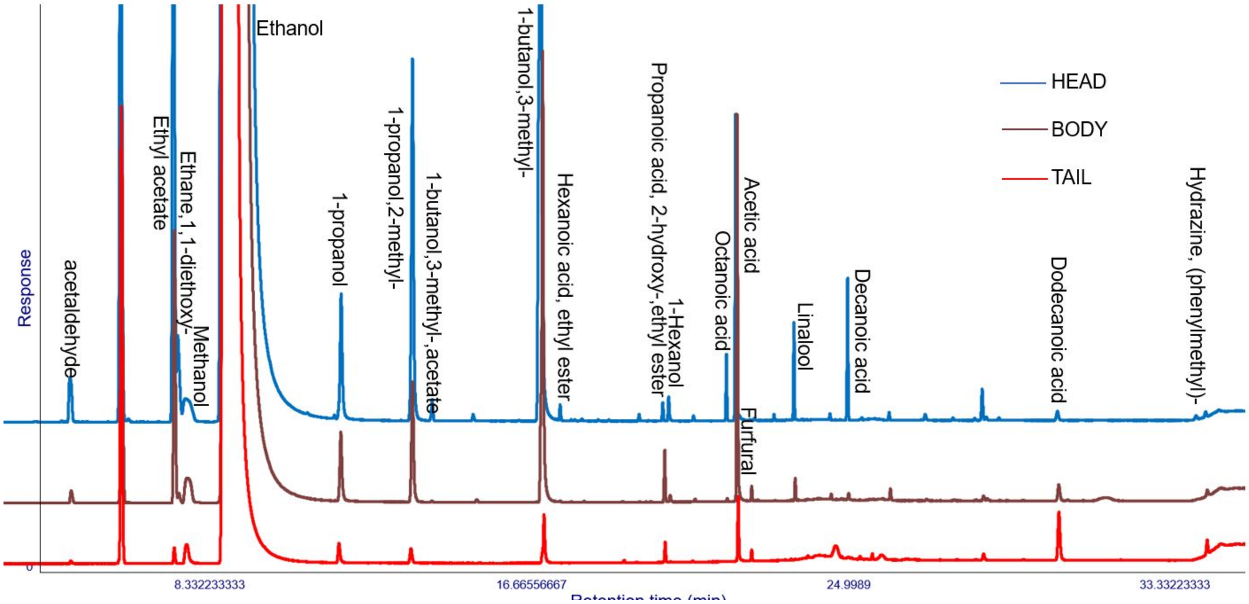

Figure A1: representative gas chromatograms of head, body, and tail of Pisco distillates
